# MADS-box gene *AaSEP4* promotes artemisinin biosynthesis in *Artemisia annua*

**DOI:** 10.3389/fpls.2022.982317

**Published:** 2022-08-31

**Authors:** Tian-Tian Chen, Xing-Hao Yao, Hang Liu, Yong-Peng Li, Wei Qin, Xin Yan, Xiu-Yun Wang, Bo-Wen Peng, Yao-Jie Zhang, Jin Shao, Xin-Yi Hu, Qing Miao, Xue-Qing Fu, Yu-Liang Wang, Ling Li, Ke-Xuan Tang

**Affiliations:** Joint International Research Laboratory of Metabolic and Developmental Sciences, Frontiers Science Center for Transformative Molecules, Plant Biotechnology Research Center, Fudan-SJTU-Nottingham Plant Biotechnology R&D Center, School of Agriculture and Biology, Shanghai Jiao Tong University, Shanghai, China

**Keywords:** *AaSEP4*, MADS-box, CArG-box, artemisinin, *AaGSW1*

## Abstract

The plant *Artemisia annua* is well known for its production of artemisinin, a sesquiterpene lactone that is an effective antimalarial compound. Although remarkable progress has been made toward understanding artemisinin biosynthesis, the effect of MADS-box family transcription factors on artemisinin biosynthesis is still poorly understood. In this study, we identified a MADS transcription factor, AaSEP4, that was predominantly expressed in trichome. AaSEP4 acts as a nuclear-localized transcriptional activator activating the expression of *AaGSW1 (GLANDULAR TRICHOME-SPECIFIC WRKY1)*. Dual-luciferase and Yeast one-hybrid assays revealed that AaSEP4 directly bound to the CArG motif in the promoter region of *AaGSW1*. Overexpression of *AaSEP4* in *A. annua* significantly induced the expression of *AaGSW1* and four artemisinin biosynthesis genes, including *amorpha-4,11-diene synthase (ADS)*, *cytochrome P450 monooxygenase (CYP71AV1)*, *double-bond reductase 2 (DBR2)* and *aldehyde dehydrogenase 1 (ALDH1)*. Furthermore, the results of high-performance liquid chromatography (HPLC) showed that the artemisinin content was significantly increased in the *AaSEP4-*overexpressed plants. In addition, RT-qPCR results showed that *AaSEP4* was induced by methyl jasmonic acid (MeJA) treatment. Taken together, these results explicitly demonstrate that AaSEP4 is a positive regulator of artemisinin biosynthesis, which can be used in the development of high-artemisinin yielding *A. annua* varieties.

## Introduction

Malaria is a mosquito-borne infectious disease that became a life-threatening problem with more than three billion people, especially in South-East Asia and Africa (Garcia, 2010; Ma et al., 2020). Artemisinin, a sesquiterpene lactone endoperoxide, is isolated from the traditional Chinese medicinal plant *Artemisia annua* (Zhang et al., 2014; Xiong and Huang, 2021). According to the World Health Organization (WHO), Artemisinin-based combination therapy (ACT) is considered the most recommended treatment to *Plasmodium falciparum* malaria (White, 2008). In addition, recent studies have reported that Artemisinin is also effective in the treatment of several cancers (Efferth, 2017). In yeast, semi-synthetic high production of artemisinin has been successfully developed (Paddon et al., 2013). Although production of artemisinin is quite low (0.1%–1.0% DW of *A. annua*), *A. annua* is the only plant source for artemisinin production (Shen et al., 2016). Therefore, improving the content of artemisinin in *A. annua* is necessary and urgent. The artemisinin biosynthesis pathway has been studied extensively and genes underlying most of the biosynthetic steps have been identified in *A. annua*. ADS catalyzes farnesyl diphosphate (FPP) converses to amorpha-4, 11-diene is the first step of artemisinin production, then amorpha-4, 11-diene is converted to dihy-droartemisinic acid (DHAA) through the function of CYP71AV1, DBR2, and ALDH1. Finally, DHAA is transformed to artemisinin in the glandular trichome of *A. annua.* (Mercke et al., 2000; Teoh et al., 2006, 2009; Zhang et al., 2008). Previous studies have shown that analyzing the artemisinin biosynthesis regulatory mechanism may reveal strategies for generating large yields and high-quality artemisinin (Zhang et al., 2013; Lv et al., 2016). A number of transcription factors from various families have been found to enhance the production of artemisinin *via* up-regulating the expression of *ADS*, *CYP71AV1*, *DBR2*, and *ALDH1* (Chen et al., 2017, 2021; Ma et al., 2018; Hao et al., 2019). For instance, the WRKY transcription factor *AaGSW1 (GLANDULAR TRICHOME-SPECIFIC WRKY1)* was reported to enhance artemisinin biosynthesis by directly binding to the promoter of *CYP71AV1* (Chen et al., 2017). It also has been demonstrated that overexpression of the AP2/ERF transcription factors (TFs) such as AaERF1 and AaERF2 increases the artemisinin content through increasing the transcript levels of *ADS* and *CYP71AV1* (Yu et al., 2012). However, knowledge of the transcriptional regulatory mechanisms that control the expression of four enzyme genes in artemisinin biosynthesis is rather limited.

The MADS-box TFs that share conserved DNA-binding domain have been extensively studied in plant, animal, and fungi (Shore and Sharrocks, 1995; Smaczniak et al., 2012; Schilling et al., 2018). Recently, an increasing number of studies have shown that MADS-box TFs participate in the regulation of secondary metabolism in various plants (Vrebalov et al., 2009; Martel et al., 2011). In tomato, MADS-box TF RIN (Ripening Inhibitor) through directly regulating the expression of *PSY (phytoene synthase)* to promote the lycopene accumulation (Martel et al., 2011). Similarly, silencing of the MADS-box genes *TAGL1*, and *FUL1/2 (FRUITFULL 1/2)* significantly decreased carotenoid accumulation (Vrebalov et al., 2009; Bemer et al., 2012; Wang et al., 2014). The citrus transcription factor CsMADS6 positively modulates carotenoid metabolism by directly regulating the transcript level of *LCYb1 (Lycopene β-cyclases)* and other carotenogenic genes (Lu et al., 2018). However, few researchers have been able to identify any MADS-box TFs that are involved in the regulation of artemisinin biosynthesis in *A. annua*.

In this study, we identified a MADS-box transcription factor AaSEP4 that directly binding to the promoter of *AaGSW1*. Overexpression of *AaSEP4* obviously increased the transcript levels of *AaGSW1* and all four key enzymes (*ADS, CYP71AV1, DBR2, ALDH1*), thus enhancing the artemisinin biosynthesis in *A. annua*. In conclusion, our research reveals a novel MADS-box TF that regulates artemisinin biosynthesis, which advances our understanding of the complex transcriptional regulation of artemisinin metabolism in *A. annua*.

## Materials and methods

### Plant materials and methyl jasmonate treatment

High artemisinin content *A. annua* cultivar ‘Huhao 1’ was used in this study, which originated in Chongqing and has been developed several years in Shanghai. *Artemisia annua* and *Nicotiana benthamiana* seeds were grown in pots at 24 ± 2°C and under a 16 h light photoperiod. For MeJA treatment, 2-week-old *A. annua* plants were sprayed with 100 μM methyl jasmonate (MeJA; Sigma-Aldrich), 0.1% ethanol as a mock control treatment. Leaf samples were collected at 0, 0.5, 1.5, 3, 6, 12, and 24 h after treatment.

### RNA extraction and RT-qPCR

RNA of *A. annua* tissues and leaves was extracted using a plant RNA isolation reagent (Tiangen Biotech, Beijing, China). Trichomes were isolated from buds as previously described (Wang et al., 2009). Glass beads and a commercial cell disrupter (BioSpec Products) were used to separate trichome cells from the surface of flower buds. Then cells and tissue mixture sequentially pass through a 40 μm and a 30 μm nylon sieves and finally collected glandular trichome cells in 30 μm meshes. RNA samples were extracted using a plant RNA isolation reagent (Tiangen Biotech, Beijing, China), and the reverse transcription of complementary DNA (cDNA) was performed by using a PrimeScript RT Master Mix (Takara, Japan). The expression level of all relative genes was performed on a Roche lightercycler96 real-time PCR machine (Roche, Basel, Switzerland) and using the SuperReal PreMix Plus SYBR-Green (Tiangen Biotech, China). Each sample has three biological replicates. All the primers used are listed in Supplementary Table S1.

### Subcellular localization

The ORF of *AaSEP4* was amplified using KOD plus DNA polymerase and then cloned into the plant expression vector pHB-YFP to generate a pHB-AaSEP4–YFP fusion protein. Then the plasmid was transferred into *A. tumefaciens* strain GV3101 for *N. benthamiana* leaf transient expression. After 48 h low light condition, the fluorescent signals of *N. benthamiana* leaves were observed by confocal laser microscopy (Leica TCS SP5-II). 4′, 6-diamidino-2-phenylindole (DAPI) was used for nuclei stain, pHB-YFP was used as negative control.

### Transformation of *Artemisia annua*

The 738 bp full-length cDNA of *AaSEP4* was amplified by using KOD plus DNA polymerase (Toyobo, Osaka, Japan) and then cloned into the pHB vector. The construct pHB-ANAaSEP4 was introduced into the *Agrobacterium tumefaciens* strain EHA105 and genetically transformed into *A. annua* for further analysis as described previously (Hao et al., 2019). Firstly, *A. annua* seeds were placed on germination medium MS_0_ and then cultured at 24°C–26°C with 16 h light and 8 h dark treatment (8,000 lux). After 2 weeks, the leaves of the germinated seedlings were collected and cut into 0.5-cm-diameter discs, then these cut leaves were co-cultivated with *A. tumefaciens* strain EHA105 at 25°C for 3 days. Then the leaves were transferred to the selective medium MS_1_ (MS_0_ + 2.5 mg/L N_6_-benzoyladenine +0.3 mg/L naphthalene-1-acetic acid +50 mg/L hygromycin +250 mg/L carbenicillin), we selected the antibiotic-resistant plantlets sub-cultured three times and then transferred them into rooting medium MS_2_ (½ MS_0_ + 250 mg/L carbenicillin). Finally, the rooted plantlets were transferred to soil pots in the growth chamber after 1 month.

### Gus expression in 1391Z-pro*AaSEP4*-GUS transgenic *Artemisia annua* plants

To construct 1391Z-pro*AaSEP4*-GUS, the 1,386-bp promoter region upstream of the start codon of *AaSEP4* was amplified from the *A. annua* genomic DNA library and inserted into the pCambia1391Z vector. The plasmids 1391Z- pro*AaSEP4*-GUS were introduced into *A. annua* plants using *Agrobacterium*-mediated genetic transformation, as described previously (Hao et al., 2019). Four-week-old 1391Z-pro*AaSEP4*-GUS transgenic *A. annua* plants were stained to observe the tissue distribution. GUS assay was performed as previously described (Xie et al., 2021). GUS staining solution [1 mM 5-bromo-4-chloro-3-indolyl-β-_D_-glucuronic acid, 100 mM Na_2_HPO_4_, 50 mM KH_2_PO_4_, 10 mM Na_2_EDTA, 0.5 mM K_3_Fe(CN)_6_, 0.5 mM K_4_Fe(CN)_6_, and 0.1% (v/v) Triton X-100] was used to stain leaves, then leaves were incubated at 37°C for 12 h in the dark. After staining, 70% ethanol was used to remove chlorophyll for three times.

### Yeast one-hybrid assays

The fragment containing AaSEP4 binding site (GArG-box) was amplified from the promoter of *AaGSW1* and cloned into the pLacZ vector. The ORF of *AaSEP4* was amplified and ligated into the pB42AD vector. Various combinations of pB42AD-AaSEP4/pB42AD and pLacz-3 x CArG-box/pLacz-3 x mCArG-box were co-transformed into the yeast strain EGY48a. An empty pB42AD vector was used as a negative control. The transformed yeast cells were grown on SD/−Trp/-Ura plates at 30°C for 2–4 days. SD/−Trp/-Ura plates with X-gal were used as test media. Yeast one-hybrid assays were conducted as previously described (Zhong et al., 2018).

### Dual-LUC assay

The promoter of *AaGSW1* was cloned into pGREEN II 0800 vector as reporter and transformed into *A. tumefaciens* strain GV3101 with the helper plasmid pSoup 19. PHB-AaSEP4 was transformed into *A. tumefaciens* strain GV3101 to act as an effector and pHB empty vector was used as a negative control. The effectors and reporter were mixed in a 9:1 volume ratio to transform 4-week-old tobacco leaves (Hellens et al., 2005). The infiltrated leaves of *N. benthamiana* were detected after 48 h low light incubation by using the Dual-Luciferase Reporter Assay System (Promega, United States). The activity of LUC was normalized to the activity of REN, and the relative LUC/ REN ratios were used to represent the activity of the promoter. Four biological repeats were performed for each sample.

### Artemisinin content measurement

Leaves of 4-month-old *A. annua* were gathered and dried at 50°C in an oven. Subsequently, leaves were ground into powder and 0.1 g powder was extracted twice with 2 ml methanol under ultrasound for 30 min (55 W, 30°C). After centrifuging 12,000 *g*, 10 min, the supernatants were filtered through nitrocellulose (0.22 μm). High-performance liquid chromatography (HPLC) was used to analyze the contents of artemisinin (Qin et al., 2021). Three repeats were measured in all samples.

## Results

### Cloning and characterization of AaSEP4

**Figure 1:**
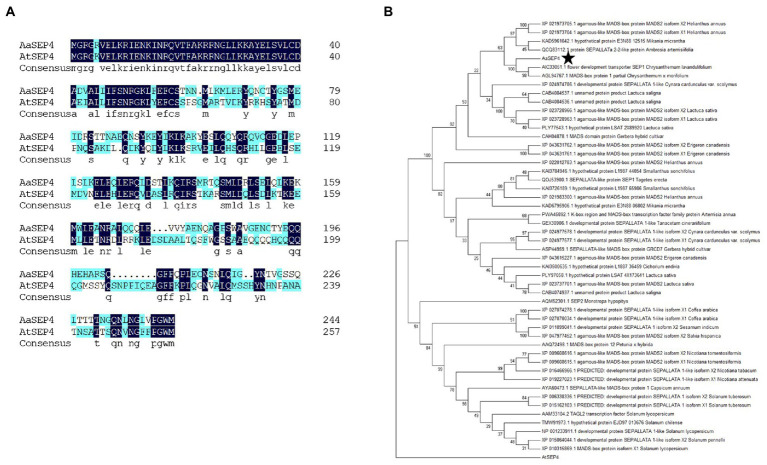
Phylogenetic analysis of AaSEP4. **(A)** The protein sequence alignment of AaSEP4 and AtSEP4. **(B)** Phylogenetic analysis was performed using MADS family proteins from various other plant species. The tree presented here is a neighbor-joining tree based on amino acid sequence alignment and constructed using the program MEGA.

AaGSW1, a glandular trichome-specific WRKY transcription factor, which is a key positive regulator of artemisinin biosynthesis in *A. annua*. To identify TFs that regulate artemisinin metabolism, we performed a yeast one-hybrid (Y1H) screen. In this study, the promoter sequences of *AaGSW1* were used as bait to screen a cDNA library derived from young leaves of *A. annua*. Several TFs were identified, one of which encoding a protein belonging to the MADS-box TF superfamily. This MADS-box TF was orthologous gene *AtSEP4* from *Arabidopsis thaliana* by a BLAST search of the TAIR database (Figure 1A). Thus, we named this MADS-box TF in *A. annua* as AaSEP4. The full-length coding sequence of *AaSEP4* encoded a protein of 245 amino acids with a calculated molecular mass of 28.36 kDa and a predicted pI of 8.15.^1^ To further understand the relationship of AaSEP4 to other MADS proteins, a neighbor-joining tree of AaSEP4 and other MADS-box family members in different plant species was constructed (Figure 1B).

### Expression profile of *AaSEP4*

**Figure 2:**
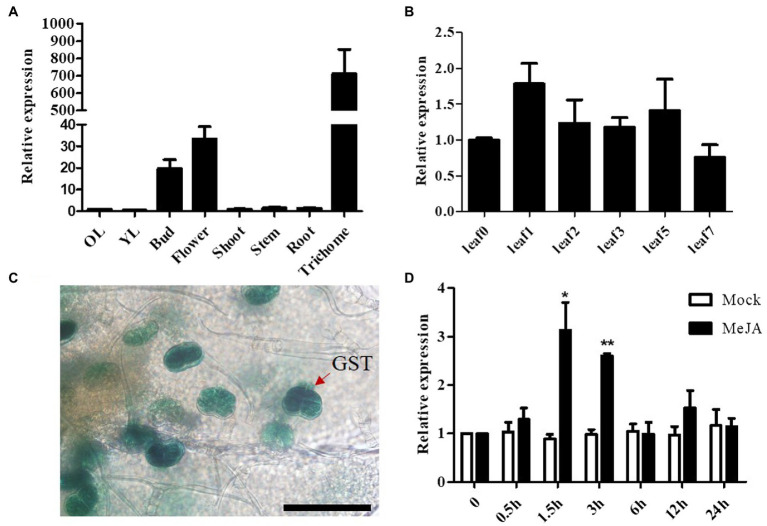
Transcript levels of *AaSEP4* in *Artemisia annua*. **(A, B)** Relative expression levels of *AaSEP4* in different tissues **(A)** and at different stages of leaves **(B)**. OL, old leaves; YL, young leaves. Data values are means ± SD (*n* = 3). **(C)** β-Glucuronidase expression of 1391-pro*AaSEP4*-GUS transgenic *A. annua* plants. Bars: 100 μm. **(D)** Relative expression of *AaSEP4* in response to methyl jasmonate (MeJA, 100 μM) by RT-qPCR. Plants were treated with ddH_2_O as mock. All data are given as means ± SD (*n* = 3) ^*^p < 0.05; ^**^p < 0.01; Student’s *t*-test.

To understand the spatial and temporal expression patterns of *AaSEP4,* we determined its relative transcript levels in different tissues and during the different stages of leaf development in *A. annua* by RT-qPCR. As Figure 2A shown, *AaSEP4* was predominantly expressed in the trichome, flower and bud. During leaf development, the transcript levels of *AaSEP4* showed no obvious difference (Figure 2B). Furthermore, 2,186 bp sequence of the *AaSEP4* promoter was amplified and generated the construct 1391Z-pro*AaSEP4*-GUS, then transformed it into *A. annua*. The GUS staining was strongly detected in the glandular secretary trichome (GST) of the transgenic plants (Figure 2C). Previous reports showed that artemisinin biosynthesis is promoted by JA and the expression of *AaGSW1* was significantly increased after JA treatment (Zhang et al., 2015; Chen et al., 2017). We therefore investigated whether JA regulates *AaSEP4* expression. The results of RT-qPCR experiments revealed that the expression of *AaSEP4* was induced drastically increased after 1.5 h of MeJA treatment compared to that in the mock-treated leaves (Figure 2D). These results indicated that *AaSEP4* has potentially function in the GST of *A. annua* and was induced by MeJA treatment.

### Subcellular localization of AaSEP4

**Figure 3:**
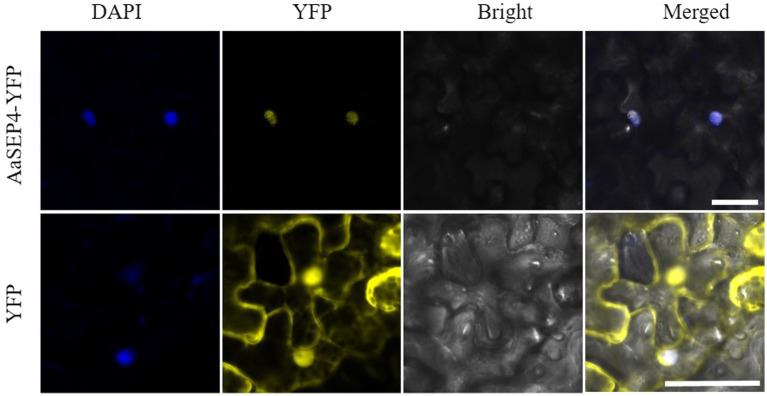
The subcellular localization of AaSEP4 in leaves of *N. benthamiana.* Yellow, yellow fluorescent protein (YFP). Blue, 4′, 6-diamidino-2-phenylindole staining (DAPI). Bars, 50 μm.

To further explore the subcellular localization of AaSEP4, we generated a pHB-AaSEP4-YFP (yellow fluorescent protein) fusion construct and transiently expressed in *N. benthamiana* leaves (Figure 3). Using fluorescence microscopy, we found that the YFP signals exceptionally in the nucleus and overlapped extensively with the DAPI signals. These data indicated that AaSEP4 protein localized to the nucleus, which is consistent with its role as a TF.

### AaSEP4 directly binds to and activates the promoter of *AaGSW1*

**Figure 4:**
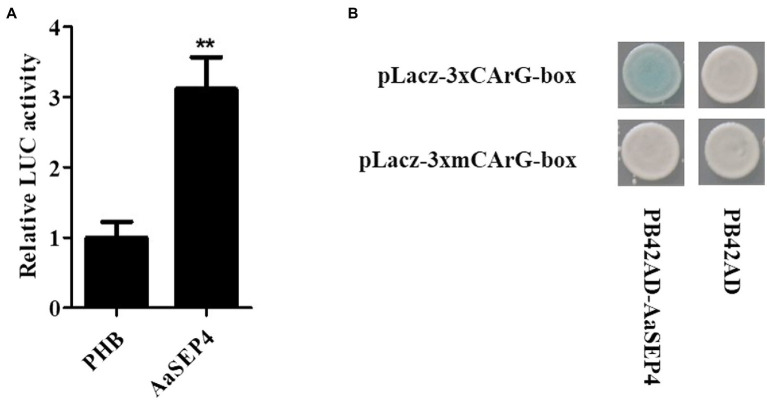
AaSEP4 directly binds and activates the promoter of *AaGSW1*. **(A)** Transient dual-LUC detected in tobacco leaves. Effects of AaSEP4 on *AaGSW1* promoter activation. The relative LUC activity was normalized to the reference Renilla (REN) luciferase. Error bars indicate SD (*n* = 3). Student’s *t*-test: ^**^p < 0.01. **(B)** Yeast one-hybrid assay of AaSEP4 and GArG-box motif in promoter of *AaGSW1*. Empty vector pB42AD was used as a negative control.

To test the interaction between the AaSEP4 protein and the *AaGSW1* promoter as previously described, we first performed a dual-luciferase assay. As shown in Figure 4A, compared with the control (empty PHB vector), the relative luciferase expression driven by the promoter of *AaGSW1* was significantly higher in the presence of AaSEP4. This result suggests that AaSEP4 activated the promoter activity of *AaGSW1*. Plant MADS-box proteins bind to specific DNA sequences known as CArG element sequence 5′-CC(A/T)6GG-3′ (Smaczniak et al., 2012; Käppel et al., 2018; Li et al., 2019), we found one CArG-box by analyzing the *AaGSW1* promoter (Supplementary Figure S1). To further confirm the binding activity of AaSEP4 on *AaGSW1* promoter, Y1H assay was performed to test if AaSEP4 could bind to this motif. As Figure 4B shown, AaSEP4 bound to CArG-box motif in the promoter region of *AaGSW1*. Taken together, these results indicated that AaSEP4 protein activated the promoter activity of *AaGSW1* by interacting with the CArG element in the promoter region of *AaGSW1.*

### Overexpression of *AaSEP4* in *Artemisia annua* increases artemisinin biosynthesis

**Figure 5:**
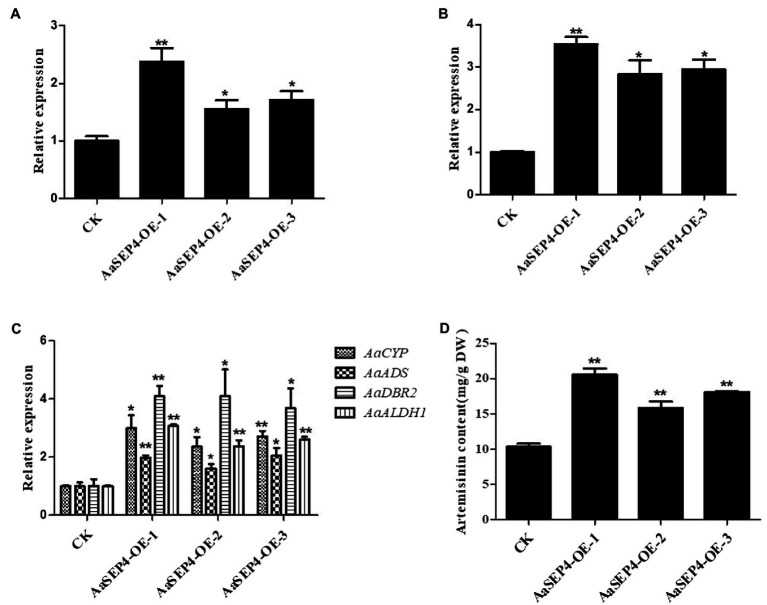
AaSEP4 is a positive regulator of artemisinin biosynthesis. Expression levels of *AaSEP4* **(A)**, *AaGSW1* **(B)** and four enzyme genes **(C)** in *AaSEP4* overexpression transgenic plants. *Actin* was used as internal reference. **(D)** Artemisinin content in *AaSEP4* overexpression lines measured by high-performance liquid chromatography (HPLC). All data are given as means ± SD (*n* = 3) ^*^p < 0.05; ^**^p < 0.01; Student’s *t*-test.

Since AaSEP4 activated *AaGSW1* directly, we further explored the role of AaSEP4 in the artemisinin biosynthesis. According to the results of RT-qRCR, we selected three representative transgenic lines (AaSEP4-OE-1, AaSEP4-OE-2, AaSEP4-OE-3) that accumulated high levels of *AaSEP4* transcript for further study (Figure 5A). In AaSEP4-overexpressing lines, the transcript level of *AaGSW1* was significantly increased by 2–3 times (Figure 5B), as well as the transcript levels of *ADS*, *CYP71AV1*, *DBR2* and *ALDH1* (Figure 5C). In addition, HPLC was used to measure the artemisinin content of 5-month-old AaSEP4-overexpressing transgenic *A. annua*. It was found that the artemisinin content of AaSEP4-OE lines was 19%–72% higher than that in the WT *A. annua* (Figure 5D). These results demonstrated that AaSEP4 positively regulates the artemisinin biosynthesis by up-regulating the transcription level of *AaGSW1* and four enzyme genes of the artemisinin biosynthesis.

## Discussion

Artemisinin is the key component of artemisinin-based combination therapies (ACTs) for malaria (Talman et al., 2019). Although the production of artemisinin is quite low (0.1%–1.0% DW), Chinese traditional herb *A. annua* is the main source to extract artemisinin currently (Hao et al., 2019). The biosynthetic pathway of artemisinin has been elucidated in depth. Dissection of the regulatory mechanism of artemisinin in *A. annua* is an effective strategy to improve the artemisinin production. Several transcription factors families such as TCP, AP2/ERF, bHLH and WRKY have been reported to regulate artemisinin biosynthesis by directly or indirectly increasing the transcript levels of four key enzyme genes in *A. annua* (Xiang et al., 2019; Fu et al., 2021; Ma et al., 2021; Wu et al., 2021). For better understanding of the mechanisms regulating artemisinin metabolism, we identified a MADS-box TF AaSEP4 that with potential roles in regulating the expression of *AaGSW1* and accumulation artemisinin in this study. AaSEP4 belongs to the AGAMOUS-like subfamily and is homologous to the AtSEP4 protein from *Arabidopsis* (Figure 1). In addition, we found that *AaSEP4* was strongly expressed in glandular secretary trichomes where the artemisinin is mainly synthesized and stored in *A. annua* (Figure 2). Using Y1H and dual-luciferase assays, we firstly demonstrated that AaSEP4 directly bound to the promoter of *AaGSW1* and activated its promoter activity (Figure 4). In addition, the transcript levels of *AaGSW1* were higher in AaSEP4-overexpressing *A. annua* plants than those in the control (Figures 5A,B). The expression levels of *ADS*, *CYP71AV1*, *DBR2*, and *ALDH*1 were also strongly induced in *AaSEP4*-overexpressed plants when compared with control plant (Figure 5C). These results are consistent with previous results that AaGSW1 directly activates *CYP71AV1* promoter *in vivo* and promotes *ADS*, *DBR2* and *ALDH1* expression indirectly (Chen et al., 2017). There is no doubt that the artemisinin content was significantly enhanced in *AaSEP4*-overexpressed plant as Figure 5D shown.

Previous studies have reported that several MADS-box TFs bind to promoters and directly regulate the transcription of their target genes, then affect the related metabolites accumulation (flavonoid carotenoid, lycopene; Wang et al., 2014; Lu et al., 2018; Li et al., 2019). In this study, we identified for the first time a MADS-box TF AaSEP4 that are involved in the regulation of artemisinin metabolism. Although AaSEP4 can only activate the promoter of *AaGSW1*, the expression of *ADS*, *CYP71AV1*, *ALDH1* and *DBR2* were also altered by the overexpression of *AaSEP4*. In addition, *AaSEP4* was significantly induced by MeJA treatment (Figure 2D). The transcriptional regulation of artemisinin metabolism in *A. annua* is complex and how *AaSEP4* regulates the artemisinin metabolism through JA signaling need to be further explored. This study demonstrates that the transcription factor AaSEP4 functions positively in the artemisinin promotion and provides insight into the engineering of artemisinin biosynthesis in the future.

## Data availability statement

The original contributions presented in the study are included in the article/Supplementary material, further inquiries can be directed to the corresponding author.

## Author contributions

T-TC and K-XT designed the project. T-TC, Y-PL, WQ, X-QF, Q-M, X-Y, X-YW, and Y-JZ performed most of the experiments. B-WP, HL, LL, X-HY, JS, X-YH, Y-LW, and K-XT analyzed the data and discussed the article. T-TC wrote the manuscript. All authors contributed to the article and approved the submitted version.

## Funding

This work was supported by the National Key R&D Program of China (2018YFA0900600), the Shanghai Science and Technology Innovation Action Plan (19431901700), the Bill & Melinda Gates Foundation (OPP1199872 and INV- 027291), SJTU Trans-med Awards Research (20190104) and the SJTU Global Strategic Partnership Fund (2020 SJTU-CORNELL).

## Conflict of interest

The authors declare that the research was conducted in the absence of any commercial or financial relationships that could be construed as a potential conflict of interest.

## Publisher’s note

All claims expressed in this article are solely those of the authors and do not necessarily represent those of their affiliated organizations, or those of the publisher, the editors and the reviewers. Any product that may be evaluated in this article, or claim that may be made by its manufacturer, is not guaranteed or endorsed by the publisher.

## Footnotes

^1^ http://web.expasy.org/protparam/
