# Supplementary material for: MADS-box gene AaSEP4 promotes artemisinin biosynthesis in Artemisia annua
Source: Front Plant Sci. 2022 Aug 31;13:982317. doi: 10.3389/fpls.2022.982317 (PMC9473666; doi:10.3389/fpls.2022.982317)
Supplement: Supplementary file 1 [file Data_Sheet_1.docx]

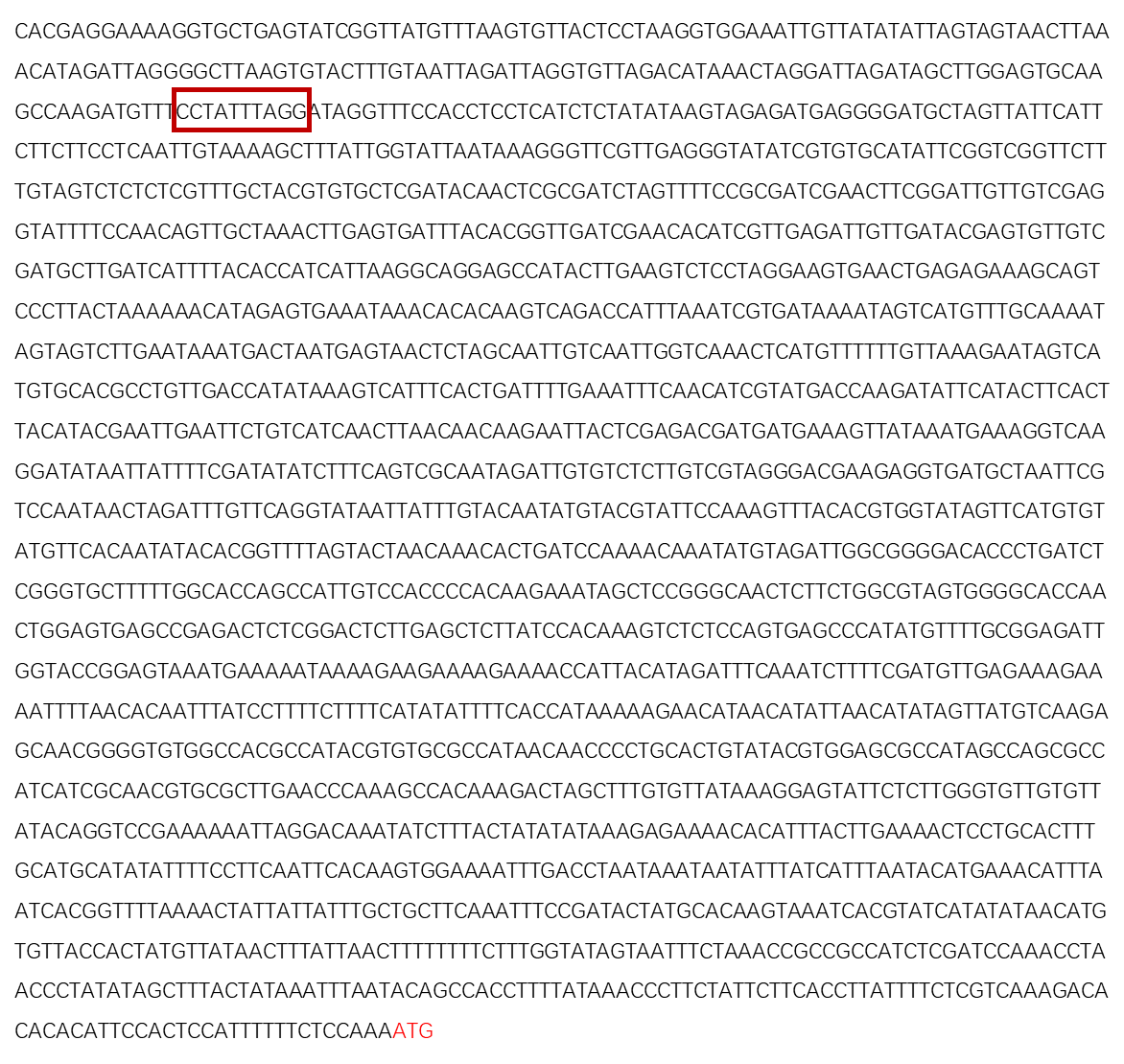


Fig.S1. Promoter sequence of *AaGSW1*

Red box; CArG motif

Table S1. All primers used in this study

| Description | Primers | 5’-3’ |
| --- | --- | --- |
| pB42AD-AaSEP4-F | Forward | GCCTCTCCCGAATTCATGGGTAGGGGAAGAGTGGA |
| pB42AD-AaSEP4-R | Reverse | CCAAAGCTTCTCGAGTCAAAGCATCCATCCAGGGA |
| placz-pro*AaGSW1*-3xCArG-F | Forward | aattcTGTTTCCTATTTAGGATAGGTGTTTCCTATTTAGG ATAGGTGTTTCCTATTTAGGATAGGc |
| placz-pro*AaGSW1*-3xCArG-R | Reverse | tcgagCCTATCCTAAATAGGAAACACCTATCCTAAATAG  GAAACACCTATCCTAAATAGGAAACAg |
| placz-pro*AaGSW1*-3xmCArG-F | Forward | AATTCTGTTTCCTATCGAGGATAGGTGTTTCCTATCGAGG ATAGGTGTTTCCTATCGAGGATAGGC |
| placz-pro*AaGSW1*-3xmCArG-R | Reverse | TCGAGCCTATCCTCGATAGGAAACACCTATCCTCGATAG  GAAACACCTATCCTCGATAGGAAACAG |
| pGreen-pro*CYP*-F | Forward | ggatccAATGGGTCAATTTCGGG |
| pGreen-pro*CYP*-R | Reverse | ccatggTGCTTTTAGTATACTCTTCA |
| pGreen-pro*ALDH1*-F | Forward | CGGTATCGATAAGCTTATGAACCATTAGAAGGGAAG |
| pGreen-pro*ALDH1*-R | Reverse | ATCCCCCGGGCTGCAGCTTTGTTTTTTATGAAATTT |
| pGreen-pro*ADS*-F | Forward | ggatccGTATTAGGGCACCAAACATCAA |
| pGreen-pro*ADS*-R | Reverse | ccatggGATTTTACAAACTTTGAATA |
| pGreen-pro*DBR2*-F | Forward | ggatccGGGAACTAACAAGATCCACACA |
| pGreen-pro*DBR2*-R | Reverse | ccatggTATTGAGTTTGATGTTGACCAGG |
| 1391Z-pro*AaSEP4*-F | Forward | CAGGTCGACGGATCCCGAGTTTTTAGCAAGATTAG |
| 1391Z-pro*AaSEP4*-R | Reverse | TCAGATCTACCATGGTTTTCACAATTTTTAAGATA |
| *Actin*-F | Forward | CCAGGCTGTTCAGTCTCTGTAT |
| *Actin*-R | Reverse | CGCTCGGTAAGGATCTTCATCA |
| qPCR-*AaSEP4*-F | Forward | GCAACAACAGCTGGAAGTAG |
| qPCR-*AaSEP4*-R | Reverse | TTGTTAACTGACTTGAGCC |
| pHB-AaSEP4-YFP-F | Forward | TCTAAGCTTGGATCCATGGGTAGGGGAAGAGTGGA |
| pHB-AaSEP4-YFP-R | Reverse | GCTCACCATACTAGTAAGCATCCATCCAGGGACTA |
| pGreen-pro*GSW1*-F | Forward | ggatccCACGAGGAAAAGGTGCTGAGT |
| pGreen-pro*GSW1*-R | Reverse | ccatggTTTGGAGAAAAAATGGAGTGG |
| pGreen-LUC-R | Reverse | AGCGTAAGTGATGTCCACCTCG |
| qPCR-*ADS*-F | Forward | GGACTAGGTTCAGGCTATG |
| qPCR-*ADS*-R | Reverse | GGACTAGGTTCAGGCTATG |
| qPCR-*CYP*-F | Forward | TCATTTCAGTCGCTT |
| qPCR-*CYP*-R | Reverse | CCAGTTTGCCTCAGTA |
| qPCR-*DBR2*-F | Forward | ACTGCTGGTGGCTTTCTTA |
| qPCR-*DBR2*-R | Reverse | ACCCTCGACTTGTTCCTTA |
| qPCR-*ALDH1*-F | Forward | GGACTTGCCTCAGGTGTAT |
| qPCR-*ALDH1*-R | Reverse | GTGCCTCTAATCCTTGTTC |
| qPCR-*AaGSW1*-F | Forward | TCTCGTCAAAGACACACACATTC |
| qPCR-*AaGSW1*-R | Reverse | TTGTTCGTAGTTGCTGTAGTGCT |
